# Brain Lesions among Orally Fed and Gastrostomy-Fed Dysphagic Preterm Infants: Can Routine Qualitative or Volumetric Quantitative Magnetic Resonance Imaging Predict Feeding Outcomes?

**DOI:** 10.3389/fped.2017.00073

**Published:** 2017-04-10

**Authors:** Nasser H. Kashou, Irfaan A. Dar, Mohamed A. El-Mahdy, Charles Pluto, Mark Smith, Ish K. Gulati, Warren Lo, Sudarshan R. Jadcherla

**Affiliations:** ^1^Wright State University, Image Analysis Lab, Dayton, OH, USA; ^2^Innovative Research Program in Neonatal and Infant Feeding Disorders, The Research Institute at Nationwide Children’s Hospital, Columbus, OH, USA; ^3^Department of Radiology, Nationwide Children’s Hospital, Columbus, OH, USA; ^4^Division of Neonatology, Department of Pediatrics, The Ohio State University College of Medicine, Columbus, OH, USA

**Keywords:** infant, volumetric, cerebellum, dysphagia, segmentation, feeding

## Abstract

**Introduction:**

The usefulness of qualitative or quantitative volumetric magnetic resonance imaging (MRI) in early detection of brain structural changes and prediction of adverse outcomes in neonatal illnesses warrants further investigation. Our aim was to correlate certain brain injuries and the brain volume of feeding-related cortical and subcortical regions with feeding method at discharge among preterm dysphagic infants.

**Materials and methods:**

Using a retrospective observational study design, we examined MRI data among 43 (22 male; born at 31.5 ± 0.8 week gestation) infants who went home on oral feeding or gastrostomy feeding (G-tube). MRI scans were segmented, and volumes of brainstem, cerebellum, cerebrum, basal ganglia, thalamus, and vermis were quantified, and correlations were made with discharge feeding outcomes. Chi-squared tests were used to evaluate MRI findings vs. feeding outcomes. ANCOVA was performed on the regression model to measure the association of maturity and brain volume between groups.

**Results:**

Out of 43 infants, 44% were oral-fed and 56% were G-tube fed at hospital discharge (but not at time of the study). There was no relationship between qualitative brain lesions and feeding outcomes. Volumetric analysis revealed that cerebellum was greater (*p* < 0.05) in G-tube fed infants, whereas cerebrum volume was greater (*p* < 0.05) in oral-fed infants. Other brain regions did not show volumetric differences between groups.

**Conclusion:**

This study concludes that neither qualitative nor quantitative volumetric MRI findings correlate with feeding outcomes. Understanding the complexity of swallowing and feeding difficulties in infants warrants a comprehensive and in-depth functional neurological assessment.

## Introduction

Contribution from several cortical and subcortical structures and various muscles and bones of the face and neck is crucially important to achieve normal feeding process (1). Organization and activation of neural centers involved in feeding process is very complex and require coordination among multiple cortical and subcortical areas. Functional neural circuitry related to infant feeding, characterization of specific neuromotor mechanisms of oral bolus transit and the influence of the cortical or subcortical regions during development of feeding skills have been reported, yet warrant further delineation (2–6).

Cranial nerves, brainstem nuclei, and cerebellum play critical subcortical roles, while cortical involvement particularly the frontal operculum, pre-frontal cortex, basal ganglia, thalamus, and insula are especially important in the voluntary control of deglutition. The thalamus and basal ganglia house motor neurons connecting the somatosensory cortex with the brainstem allowing for cortical input to and modulation of the swallowing processes (1, 7). The cerebellum has been shown to provide a regulatory component to the swallowing process, modulating cortical output of excitation or inhibition (8). The brainstem contains the central pattern generator and nerve groups important to the swallowing function, with key components residing in the medulla and midbrain (9). Six of the 12 cranial nerves (V, VII, IX, X, XI, and XII), emanate from the pons and medulla, are involved in various aspects of oral-, pharyngeal-, and esophageal-phases of swallowing, with functions ranging from conveying taste sensation to the control of orofacial and foregut musculature (1).

Impairment occurring at neural centers involved in feeding process could potentially cause difficulty with feeding and/or swallowing known as dysphagia, wherein bolus flow is either stopped in the oropharynx or misdirected into the airway (10, 11). If the dysphagia is severe, a gastrostomy tube (G-tube) will be required to deliver nutrition and hydration (7, 11). Infants discharged with a G-tube will have a long-term morbidity risk related to enteral tubes, compared to orally fed infants. G-tube may also result in an aversion to feeding and increased hospital admittance, which will lead to socioeconomic burdens on families and society (12–16). Sensitive tools that can early identify brain anatomical changes and predict subsequent neurodevelopmental disability are crucially important for early intervention and amelioration of potential problems.

Magnetic resonance imaging (MRI) provides a safer alternative to computed tomography imaging of neonates (17). The subsequent development of qualitative MRI further advanced the neuroanatomical studies of the brain structure in adults and infants. Neuroanatomical MRI has widely been used to detect varieties of white-matter (WM) and gray matter (GM) abnormalities, including signal abnormalities, loss of volume, enlarged ventricles, thinning of the corpus callosum, and delayed myelination in preterm and term infants (18–22). Functional MRI (fMRI) has been used to examine neural activation patterns during swallowing, implicating the involvement of different brain regions in the process (1, 23–28). Brain abnormalities, especially when severe, are readily identifiable on conventional T1 and T2 weighted MRI as early as 36 to 40 weeks postmenstrual age (29, 30) and have been correlated with neurodevelopmental outcomes later in life (30–36).

Information from advanced quantitative MRI or volumetric MRI has been used to clarify brain lesions with neurodevelopmental outcomes by overcoming the limitations of qualitative MRI (33, 34, 37–39). Volumetric analysis of MRIs has intensively been used as a surrogate endpoint in high-risk newborn such as highlighting smaller regional areas of the brain and gray-matter–white-matter (GW–WM) differences of infants after treatments such as hypothermia cooling and corticosteroids as well as smaller volumes for preterm infants suffering periventricular leukomalacia (22, 38, 40).

Although organization and activation of multiple cortical and subcortical areas of the brain have been characterized and established to be involved in various aspects of oral-, pharyngeal-, and esophageal-phases of swallowing, quantitative volumetric analysis of those brain areas in dysphagic preterm infants have not been delineated. We designed a retrospective study to test the hypothesis that early performance of volumetric MRI analysis of cortical and subcortical brain regions would provide more insights on the outcome of feeding process in dysphagic infants. Our aim was to evaluate whether volumetric analysis may be used as a sensitive tool to: (1) detect regional brain abnormalities in infants with feeding disorder; (2) determine whether differences of different brain regions’ volume correlate with, and may correlate with feeding outcomes of dysphagic infants at discharge.

## Materials and Methods

### Subjects and Study Design

Using a retrospective design, we analyzed brain MRIs from 43 preterm infants (22 males; born at 31.5 ± 0.8 week gestation) and correlated structural findings with their functional feeding outcomes at discharge. This data were divided into two groups according to feeding outcomes at discharge: full oral feeders (*N* = 19) and G-tube feeders (*N* = 24); infants did not have G-tube at the time of the study. We excluded data from infants with genetic or congenital anomalies. After arrival to our level 4 tertiary care unit, infants were referred to our feeding program for consultation, evaluation, and management of feeding issues. MRI was done during this evaluation. Infants stay till discharge and those who fail to acquire feeding success, get a gastrostomy placed before discharge. After hospital discharge, these infants were followed by regional outpatient interdisciplinary clinics including feeding specialists, pediatric neurologists, developmental specialists, neonatologist, dietician, physical, and occupational therapists. Our program does not have access to this information after hospital discharge. All infants were in their transitional stage of feeding (both oral and gavage) during the study. Approval from the Institutional Research Review Board at Nationwide Children’s Hospital, Columbus, OH, USA was obtained to perform this investigation.

### MRI Data Acquisition Protocol

Scans were performed on naturally sleeping infants using 1.5 and 3 T (T) GE clinical scanners (General Electric Healthcare, Wisconsin, USA). MRI data consisted of both T1 and T2 weighted scans, with the following parameters (1). Axial T1 fluid attenuated inverse recovery (FLAIR): echo time (TE) = 8.5 ms, repetition time (TR) = 2,000 ms, inversion time (TI) = 750 ms, field of view = 18 cm, matrix = 256 × 256, average number of slices = 30, slice thickness = 4 mm, and scan duration = 3 min (2). Axial T2 fast spin echo (FSE): TE = 102 ms, repetition time (TR) = 4,000 ms, FOV = 18 cm, matrix = 256 × 256, average number of slices = 30, ST = 4 mm, and scan duration = 2 min and 32 s (3). Sagittal T1 FLAIR: TE = 8.5 ms, TR = 2,000 ms, TI = 750 ms, FOV = 18 cm, matrix = 256 × 256, average number of slices = 30, ST = 4 mm, and scan duration = 3 min.

### MRI Segmentations Protocol

**Figure 1:**
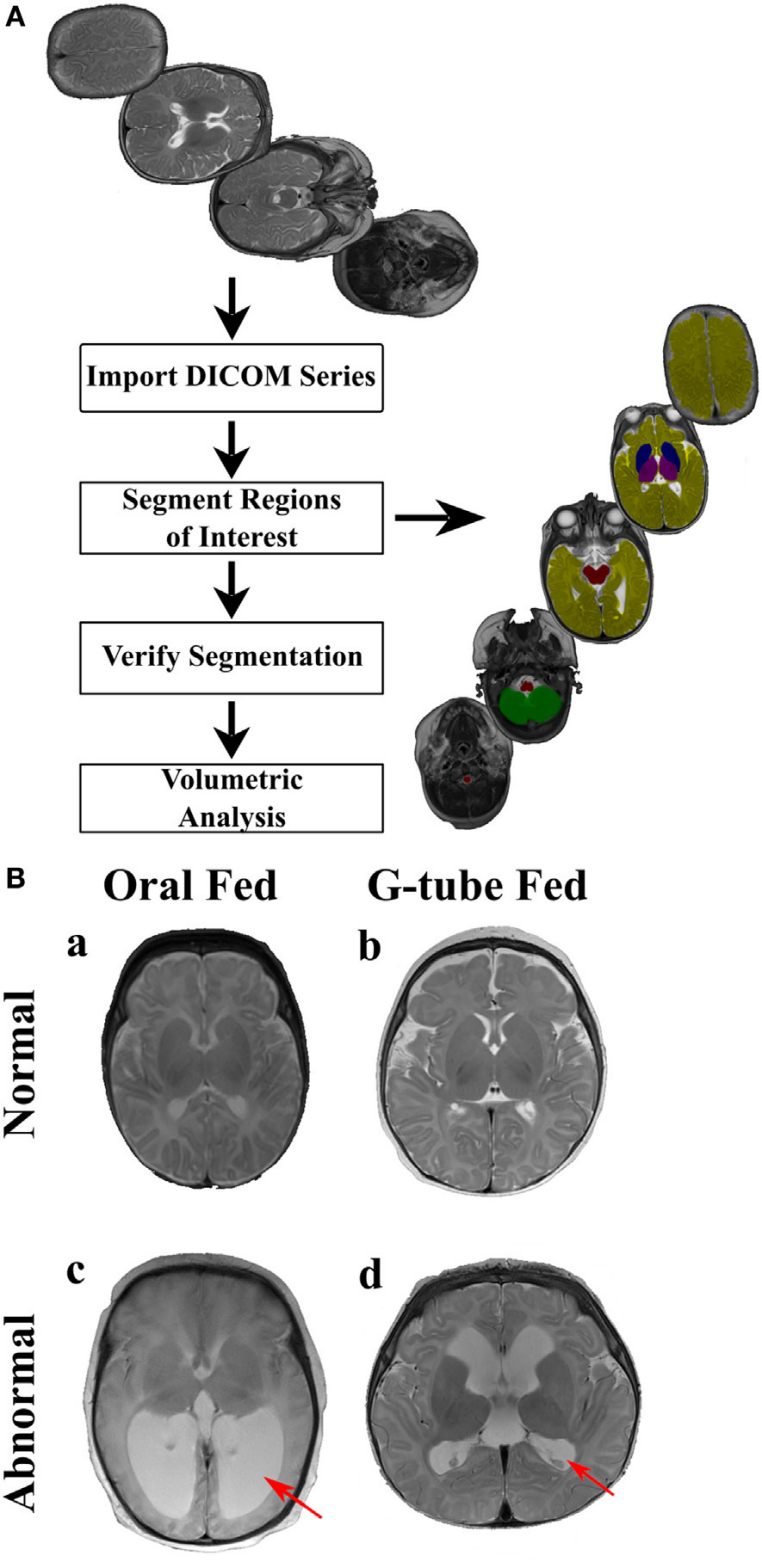
**Manual segmentation process for volumetric analysis**. **(A)** The flow chart of the segmentation process. MR images (top image) are uploaded into Analyze 12.0 software, segmented into specific regions of interest (right image) using the manual segmentation tool, segmented regions were verified and volumetric data for each segmented region was automatically computed. Segmented areas (right image) show: brainstem (red), cerebellum (green), cerebrum (yellow), basal ganglia (blue), and thalamus (purple). **(B)** Representative MR images of (a) an infant discharged on oral feeds and had a normal magnetic resonance imaging (MRI) scan, (b) an infant discharged on a G-tube and had a normal MRI scan, (c) an infant discharged on oral feeds and was diagnosed with hydrocephalus, (d) an infant discharged on a G-tube and was diagnosed with hydrocephalus. Hydrocephalus (c,d) is characterized by enlargement of lateral ventricles (red arrows), compared to normal MRIs. This figure shows independence between MRI findings and feeding outcome.

Magnetic resonance imaging sequences were processed utilizing Analyze 12.0 (AnalyzeDirect, Overland Park, KS, USA), a visualization, and analysis software tool for medical imaging. Due to heterogeneity of neonatal brain structures and for consistency with evaluation, axial T2 scans were segmented manually. Any MRI scans showing motion artifacts were removed. Segmentation was started from the most distal slice and progressing superiorly, selecting specific regions of interest (ROI) to study basal ganglia, cerebellum, brainstem, thalamus, and cerebral cortex. To adjust for age at the time of scan, volume of each ROI was calculated by summing all voxels contained in each segment and presented in relation to the whole brain volume. A flow chart of segmentation process describes our approach is shown in Figure 1A.

Segmented areas were identified and validated according to previous studies and by atlases (41–43). Because of our interest in feeding-related ROIs, we studied only specific segmented areas. Basal ganglia was defined as the area encompassing the striatum, globus pallidus, substantia nigra, and subthalamic nucleus. These areas were distinguishable by their supra-thalamic location in the axial plane and through their dark intensities, compared to the surrounding WM. Thalamus (hypothalamus, prethalamus, and epithalamus) was identified by its postero-inferior location, relative to the basal ganglia in the axial plane and by its lower intensity, compared to surrounding areas. Brainstem was identified based on its anterior location to the cerebellum as well as by its dark intensity, compared to the surrounding cerebrospinal fluid. Cerebellum was identified by its distinct location posterior to brain stem and inferior to cerebrum. Vermis was identified as the area located in the medial portion of cerebellum between the left and right hemispheres. Cerebrum was identified as the neural tissue above the tentorium cerebelli and excluded ventricles. Final segmentations were cross checked with a neonatal neuroradiologist, neurophysicist, and intra-rater segmentation to ensure accuracy. Neonatal neuroradiologist assessed each patient for the presence of neurological abnormalities, which ranged from germinal matrix hemorrhages, periventricular leukomalacia, posterior fossa subdural hematomas, hydrocephalus, in addition to lesions in the choroid plexus and basal ganglia. To determine if intraventricular hemorrhaging (IVH) was present, a cranial ultrasound was conducted and assessed by a trained radiologist at the hospital. IVH was classified from grade I to grade IV based on the severity of bleeding present based on the Papile classification. An example of normal and abnormal MRI is shown in Figure 1B.

### Statistical Analysis

Descriptive statistics are presented as mean ± SEM, unless otherwise indicated. Chi-squared tests were performed to determine if the presence of lesions in the brain was associated with feeding outcomes at discharge. ANCOVA was used to measure the association between regional brain volumes and feeding outcomes while addressing confounding variables such as postmenstrual age (PMA) at study and sex. *p*-Value of <0.05 was considered significant. All statistical analyses were performed using R software (version 3.1.2, R Development Core Team, 2016).

## Results

Demographic characteristics (Table 1) showed no significant difference between feeding outcome groups, with the exception of head circumference at the time of MRI scanning and discharge. At time of the MRI scan, 32 infants (74%) had a gastric tube inserted, while 11 infants (26%) were on oral feeds. Out of 43 infants, 19 (44.2%) infants were discharged on full oral feeds, while the remaining 24 (55.8%) were discharged with a G-tube. G-tube fed infants (vs. orally fed) had a greater head circumference. At time of study, 27 out of 43 (62.8%) of infants had bronchopulmonary dysplasia (BPD), 12 out of 27 (44.4%) were discharged on oral feeds, and the other 15 (55.6%) were discharged with a G-tube. One data set was removed from the study due excessive motion.

**Table 1:** **Demographic characteristics**.

Characteristics	Orally fed infants (*N* = 19)	G-tube fed infants (*N* = 24)	*p*-Value
Gestational age at birth (weeks)	31.8 ± 1.2	31.3 ± 1.0	0.73
Weight at birth (kg)	2.1 ± 0.3	1.90 ± 0.3	0.69
Head circumference at birth (cm)	29.9 ± 1.6	28.5 ± 1.3	0.53
Postmenstrual age at scanning (weeks)	41.7 ± 0.9	45.3 ± 1.8	0.08
Weight at scanning (kg)	3.6 ± 0.3	4.25 ± 0.2	0.06
Head circumference at scanning (cm)	34.7 ± 0.7	36.7 ± 0.6	0.05
Postmenstrual age at discharge (weeks)	47.2 ± 1.8	56.5 ± 3.8	0.04

*Student’s t-test showed no significant difference in demographic characteristics between the orally fed and the G-tube fed groups except in head circumference at magnetic resonance imaging study and age at discharge. Values are presented as mean ± SEM*.

Among those discharged on G-tube, the average (range) age when MRI scans were performed was 45 weeks PMA (30–68 weeks) compared to oral-fed discharges at 41 weeks PMA (35–54 weeks). To characterize the heterogeneity among ROIs, we clarified the presence of specific neurological lesions as shown in Table 2. Twenty-one infants (11 discharged on a G-tube, 10 discharged on oral feeds) were diagnosed with abnormalities on brain MRI and the remaining 22 infants were normal. Fourteen of those 21 infants (9 discharged on a G-tube and 5 discharged on oral feeds) were diagnosed with IVH ranging from grade I–IV (Mild–Severe). Infants were classified according to their IVH grade: seven (4 on G-tube) were IVH-I; three (2 on G-tube) were IVH-II; three (2 on G-tube) were IVH-III; and one (on G-tube) was IVH-IV. In addition to those diagnosed with IVH, three infants were diagnosed with periventricular leukomalacia (1 on G-tube), two infants (1 on G-tube) with hydrocephalus, one infant (on oral feeds) with a basal ganglia lesion, one infant (on G-tube) with germinal matrix hemorrhaging, and one infant (on oral feeds) with posterior fossa subdural hematoma. One patient (on G-tube) with hydrocephalus was also diagnosed with grade II IVH, and one patient (on G-tube) with choroid plexus lesion was diagnosed with grade I IVH. Description of these lesions are shown in Table 3. The infant with GMH did not have an IVH grade at time of the scan, so was kept separate from IVH classification. Eighteen of the 21 infants (85%) with abnormal MRI were diagnosed with BPD. Chi-squared analysis comparing MRI results with feeding outcome was not significant, although we were underpowered. Because of heterogeneity among subjects and lesions, correlation between severity of each injury and outcomes was not feasible.

**Table 2:** **Relationship between overall brain magnetic resonance imaging (MRI) findings and feeding outcome at discharge**.

MRI findings	Orally fed infants (*N* = 19)	G-tube fed infants (*N* = 24)
Abnormal MRI (*N* = 21)	52.6%	45.8%
Normal MRI (*N* = 22)	47.4%	54.2%

*p*-Value		0.892

*Infants presented with feeding difficulties were checked for neurological lesions with an MRI scan at an average of 43 weeks postmenstrual age (30–68 weeks). The scans were analyzed for abnormalities such as intraventricular hemorrhaging, hydrocephalus, germinal matrix hemorrhage, and periventricular leukomalacia. Chi-square test showed no correlation between MRI abnormalities and feeding outcome at discharge*.

**Table 3:** **Magnetic resonance imaging (MRI) abnormalities among orally fed and G-tube fed infants**.

MRI lesions	Orally fed infants (*N*)	G-tube fed infants (*N*)
Intraventricular hemorrhaging (IVH) Grade I	3	4^a^
IVH Grade II	1	2^b^
IVH Grade III	1	2
IVH Grade IV	0	1
Periventricular leukomalacia	2	1
Hydrocephalus	1	1^b^
Choroid plexus lesion	0	1^a^
Basal ganglia lesion	1	0
Posterior fossa subdural hematoma	1	0
Germinal matrix hemorrhages	0	1

*Description of the MRI abnormalities present for 21 infants in the study (10 discharged on oral feeds; 11 discharged on G-tube). IVH Grade is severity of IVH based on the Papile classification system. Data in columns 1 and 2 represent feeding outcome at discharge. One infant with germinal matrix hemorrhages did not have an IVH score at time of scan so was kept separate. The other 24 infants included in the study were assessed as neurologically normal based on MRI and ultrasound scans*.

*^a^One infant was diagnosed with both choroid plexus lesion and IVH Grade I*.

*^b^One infant was diagnosed with both hydrocephalus and IVH Grade II*.

**Figure 2:**
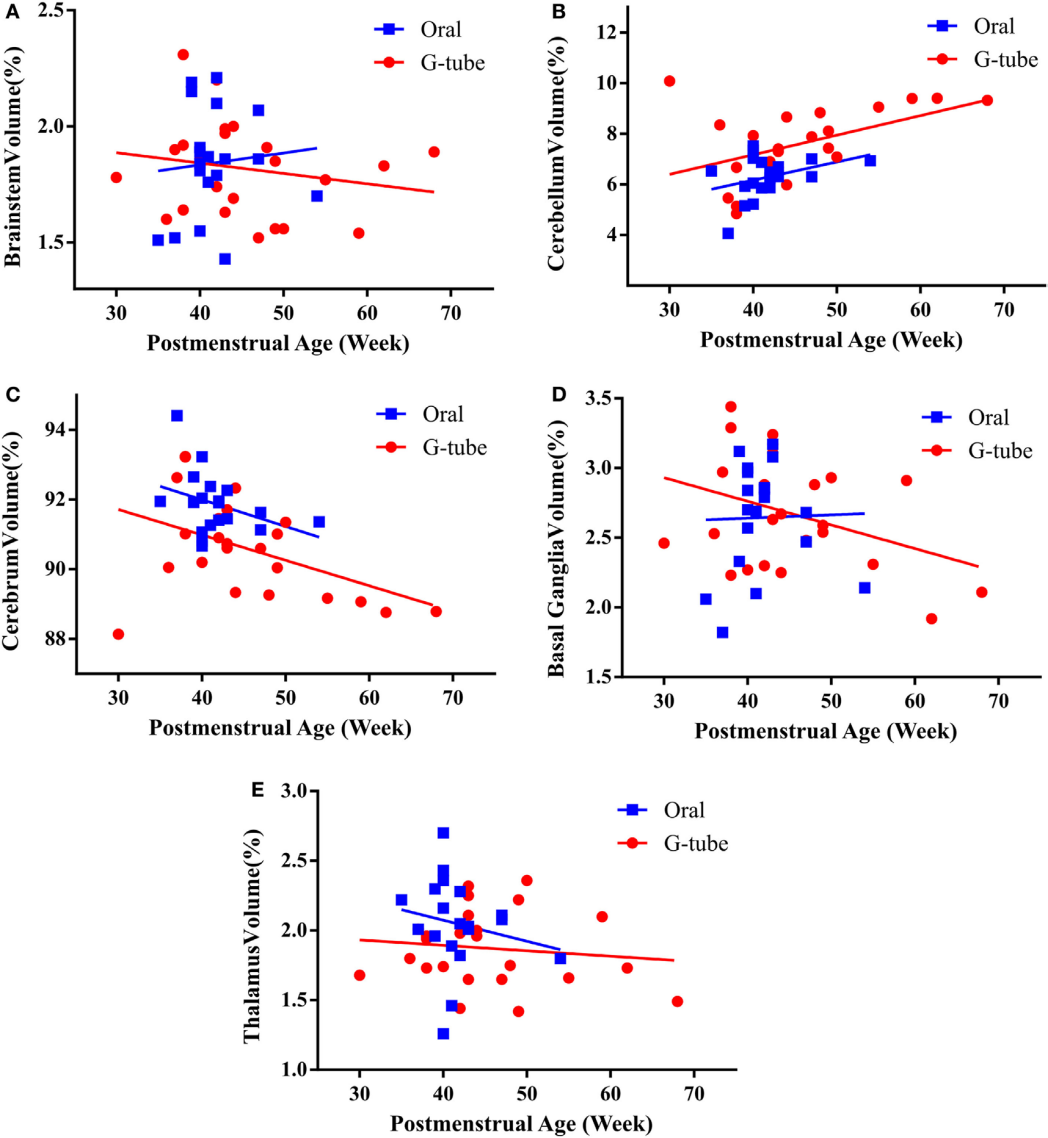
**Correlation of regional brain volumes to postmenstrual age of infants discharged on oral or G-tube feeds**. Volumes were calculated by manual segmentation of the region of interest. The ratio of regional volume to the total brain volume was calculated and correlated with postmenstrual age (PMA) for feeding outcome groups. In both feeding outcome groups, ANCOVA analysis did not show relationship between PMA and the volume of brainstem **(A)**, cerebellum **(B)**, cerebrum **(C)**, basal ganglia **(D)**, and thalamus **(E)**. Trend lines show the change in normalized volumes vs. PMA when the magnetic resonance imaging scan was taken. Regional volumes are presented as percentage of total brain volume.

We characterized the segmental ROIs (GM–WM with exclusion to ventricular volumes) as a proportion (%) of total brain volumes in relation to the PMA based on functional feeding outcomes (Figure 2). This analytical process adjusted for potential confounding factors such as head size and gender-related differences. ANCOVA of the slopes showed that there was no significant relationship of PMA or gender at MRI evaluation having any effect on the brain volume between feeding outcome groups. ANCOVA revealed that G-tube fed infants had a higher cerebellar volume (*p* < 0.05), while infants discharged on oral feeds had a higher cerebral volume (*p* < 0.05). Other ROI such as brainstem, basal ganglia, thalamus, and vermis did not show any significance between feeding outcomes (Table 4).

**Table 4:** **Volumes of different brain regions in relation to total brain volume**.

Region of interest (ROI)	Regional volume/total brain volume (% mm^3^)		*p*-Value
	Orally fed infants	G-tube fed infants	
Brainstem	1.8 ± 0.1	1.8 ± 0.04	0.74
Cerebellum	6.3 ± 0.2	7.5 ± 0.3	<0.01
Cerebrum	91.8 ± 0.2	90.7 ± 0.3	<0.01
Basal ganglia	2.6 ± 0.1	2.7 ± 0.1	0.81
Thalamus	2.0 ± 0.1	1.9 ± 0.1	0.07
Vermis	0.7 ± 0.03	0.8 ± 0.4	0.21

*Manual segmentation of specific ROI involved in the feeding pathway was done on T2 axial magnetic resonance imaging scans performed on G-tube fed infants at an average of 45 weeks postmenstrual age (PMA) (30–68 weeks) and on orally fed infants at 41 weeks PMA (35–54 weeks) using the AnalyzeDirect software. These regional volumes were normalized to total cerebral volume, to remove confounding variables such as head size and gender and presented as proportions of the total brain volume. ANCOVA showed that the volumes of different regions, except the cerebellum and the cerebrum are not significantly different in both groups. Values are presented as mean ± SEM*.

## Discussion

Impairment of neural centers involved in feeding process could potentially cause difficulty with feeding and/or swallowing (10, 11). This may result in failure to thrive and comorbidities leading to a G-tube placement (7, 11). Despite the economic burden to patients and society, brain qualitative MRI testing is routinely prescribed by clinicians to clarify structural neuropathology and possibly assign risk status for a G-tube, although it does not provide sufficient details needed for accurate diagnosis (33, 34, 37–39). Sensitive tools that can early identify brain anatomical changes and accurately detect abnormalities within individual brain regions involved in feeding/swallowing process are crucially important for early intervention and amelioration of potential problems. We designed a retrospective study to evaluate whether volumetric analysis may be used as a sensitive tool to: (1) detect neurological abnormalities in regional brain volumes of NICU infants with feeding disorder; (2) determine whether differences of different brain regions’ volume correlate with, and may predict, feeding outcomes of dysphagic infants at discharge.

In this study, we found that MRI findings did not correlate with feeding outcomes (G-tube or oral feeding). Therefore, we took a step further to examine the regional volumes of brain areas involved with feeding-related neural circuitry. We found no significant relationship of PMA or sex on volumes of brain ROIs between feeding outcome groups. Delay in acquisition of oral feeding milestones in G-tube fed infants may be a function of delayed maturation. However, there is a need for objective testing of maturational differences at neurosensory and neurocognitive functions. The only appreciable difference was that G-tube fed infants had higher head circumference and higher cerebellar volumes, while orally fed infants had a higher cerebral volume. The biological significance of this finding is not clear, and tests to assess neurosensory and neurocognitive functions related to feeding reflexes are needed.

Our observation that orally fed infants had greater cerebral volumes (as opposed to G-tube fed infants) could be explained as these infants had more oromotor feeding practice opportunities and, therefore, better regulatory ability to ingest oral nutrients safely. We speculate that these practices may have accelerated the favorable synaptogenesis vs. favorable synaptic pruning relationships in these regions. In contrast, G-tube fed infants have less oral feeding opportunities and oral stimulation may, limitedly, happen during salivation or gastroesophageal reflux events (12–16).

The precise reasons for greater cerebellar volumes among G-tube fed infants in our study are unclear. Studies testing the integrity of functional neural circuitry are needed to clarify this observation. It is known that cerebellum is the fastest growing part of the brain in the fetus and its volume increases 5-fold between 24–40 weeks gestation (44). Cerebellum plays a role in coordination of skilled motor activity and its volume is shown to be different in premature infants, compared to term infants at birth (45). Although larger cerebellum does not necessarily imply better feeding coordination in neonates, studies on infants with cerebral palsy have shown hypertrophy in neuronal tracts correlating to adjusted neuroplasticity (46). Similar to this, impairments in the brain can lead to hypertrophy in the unaffected areas, which may be analogous to our observation. The cerebellum may play a role in feedforward adaptation in swallowing pathway. By adjusting muscle exertion in response to changing environment and stimulation, successful feeding can be achieved (47). In infants, adaptations need to be made in regards to flow rate, head position, and source of food. In the clinical setting, these parameters vary from feed-to-feed and day-to-day, thus requiring constant adjustments. The cerebellum has been shown to be a key factor in motor adaptation (48, 49). In such studies, cerebellar activation was seen during adaptive split-belt treadmill experiments. Adjustment in the treadmill speed of one side demanded adaptation from the participant, which resulted in excitation in the cerebellum to provide feedforward and feedback control. In regards to swallowing, these feedforward adaptations may lead to synaptic pruning and reorganization in the cerebellum at a younger age, thus leading to a volumetric difference between oral fed and G-tube fed infants. The reorganization of the pathway may also lead to an increase in cerebral volume, as cortical regions involved with the oromotor and swallowing pathways are developed due to repetition and experience from oral feeds. This increase in cerebral volume may be attributed to the infant learning process to coordinate complex oromotor facial functions involving the tongue, jaw, face, and palate during feeding (50).

Studies have shown that volume of certain areas decreases in the presence of abnormal feeds, altered nutrition, and sensory deprivation in human (51–56) and animal studies (57, 58). The difference in cerebellar size between feeding outcomes could be a similar issue, where the volume of the segmental brain regions is correlated with the activity. Belfort et al. showed a difference in brain volume between infants that obtain majority of their nutrition through breastfeed, compared to others (59). While their study showed an increase in deep GM nuclei and ours did not, their study affirms that there is a significant difference in brain volume growth depending on nutritional intake at an early age.

Although multiple patients in our study had neurological abnormalities, certain patients were able to feed orally. A feasible comparison between abnormalities was not possible due to the low number of patients in this study, but link between lesions in GM regions of the brain as well as autism with future communication disorder and dysphagia has been proposed (7, 60). Alternatively, infants that have structural abnormalities can be free from motor impairment (cerebral palsy) later in life (30, 36, 61). This leaves the question of whether MRI alone is accurate enough to predict motor dysfunction later in life, without studying the activation of functional connectivity of swallowing and airway protection pathways (36). Some neurological injuries are subtle enough that cannot be ascertained through structural MRI scans alone (36). A similar question is posed when studying respiratory issues with infants with dysphagia (62). In Sheikh et al., the infants had no structural MRI abnormalities or congenital issues but experienced respiratory and oromotor difficulties (62). Thus, dysphagic infants may have functional irregularities such as disrupted or dysfunctional neural pathways. To ascertain these characteristics, other complementary methodologies include diffusion tensor imaging (DTI) and functional near-infrared spectroscopy (fNIRS). DTI has been used with human neonates to view microstructural abnormalities and determine if there is delayed development (63). fNIRS uses optical sources and detectors to measure the hemodynamic changes in focal regions of the cortex during functional tasks (64). Changes in oxy- and deoxy-hemoglobin correlate to neural activation in these areas, providing an alternative to fMRI. fNIRS has already been used to correlate neuronal activity during pharyngeal and esophageal swallows (65). Neurogenic dysphagia requires intensive exercise regiments (66), and the combination of these approaches may clarify the effects of therapies on integrity of structure and functions of neural circuitry.

Like any retrospective study design, our study has limitations and potential solutions can be developed from this study. (1) T2 FSE scans with a variable number of slices were used, and future studies standardizing a high slice number may be helpful for a better clarity. (2) All MRI data were standard 2-dimensional clinical scans with a lower resolution. This may have an effect on the volumetric accuracy of calculating the partial volume effects (PVE) of smaller ROIs. A 3-dimensional dataset would minimize PVE; however, it will increase scanning and processing time. Moreover, because these MRIs were not performed with very high resolution, we were restricted to make the best measurements for the sites mentioned. It is very interesting to see information on the caudate and putamen; however, we were unable to separate the putamen and caudate as this requires high quality high resolution MRIs that require prolonged scanning which can be difficult to perform on neonates. (3) Since only 21 patients had a neurological abnormality, any group comparison within the heterogeneous neuropathology was not feasible and significant observations cannot be made about feeding difficulties in relation to MRI pathology. Longitudinal scans can provide an accurate outcome assessment in regards to brain volume development and function/dysfunction. (4) Due to the low number of IVH present in our study, sub analysis between the grades of IVH were not possible, so all grades were grouped together as either a presence or absence of IVH on the ultrasound scan.

In conclusion, structural MRI is a prevalent tool in determining structural abnormalities in neonates, but current testing and analytical protocols do not provide a comprehensive analysis of any underlying neuropathology that may be the cause of neonatal feeding difficulties. Premature infants discharged on a G-tube had a higher mean cerebellar volume than infants discharged on oral feeds, while infants discharged on oral feeds had a higher mean cerebral volume, displaying a difference in neuroplasticity between feeding outcomes in neonatal dysphagia. Feeding outcomes were not dependent on presence of lesions, implying that a more comprehensive and in-depth functional neurological assessment is needed to clarify the complexity of neonatal dysphagia.

## Author Contributions

NK: co-first author, involved with concept and study design, definitions, data analysis, development of first draft, manuscript writing, and approval of final manuscript. ID: co-first author, involved with concept and study design, data and statistical analysis, development of figures and manuscript writing, development of first draft, and approval of final manuscript. ME: involved in data and statistical analysis, development of figures and manuscript writing, and approval of final manuscript. CP: involved with study design, data validation and verification of regions of interest, manuscript writing, and approval of final manuscript. MS: involved with study design, data validation and verification of regions of interest, manuscript writing, and approval of final manuscript. IG: involved with concept and study design, verification of clinical data, clinico-radiological correlations, data analysis, manuscript writing, and approval of final manuscript. WL: critical review and interpretation, manuscript writing and editing, and approval of final manuscript. SJ: principal investigator, developed concept and design, IRB process, data validation and verification of regions of interest, clinico-pathological correlations, development of first draft, manuscript writing and editing, critical review, supporting funding, project supervision, and approval of final manuscript.

## Conflict of Interest Statement

SJ is the guarantor of the article. Any potential competing interests: none.
